# Development of maxillary sinuses in relation to the development of cranium in children on computed tomography imaging

**DOI:** 10.1038/s41598-024-74586-z

**Published:** 2024-10-03

**Authors:** Przemysław Kiciński, Michał Podgórski, Beata Małachowska, Piotr Grzelak, Michał Polguj

**Affiliations:** 1 Department of Angiology, Chair of Anatomy and Histology, Medical University of Lodz, Lodz, Poland; 2 III Department of Radiology and Diagnostic Imaging, Medical University of Lodz, Lodz, Poland; 3 Department of Radiation Oncology, Albert Einstein College of Medicine, Bronx, NY USA; 4 Department of Diagnostic Imagining, Polish Mother’s Memorial Hospital Research Institute, Lodz, Poland; 5 Department of Normal and Clinical Anatomy, Chair of Anatomy and Histology, Medical University of Lodz, Lodz, Poland

**Keywords:** Maxillary sinus, Cranium, Development, Anthropometric measurement, Children, Anatomy, Musculoskeletal system, Medical research, Paediatric research

## Abstract

There are available studies assessing the development of maxillary sinuses in relation to the viscerocranium. However, there are no publications analyzing the development of maxillary sinuses in relation to the development of the cranium, i.e. both the viscerocranium and the neurocranium. The aim of the study was to analyze the correlation between the dimensions of maxillary sinuses and anthropometric measurements of the cranium in children. The study was retrospective and was conducted at the based on the results of head computed tomography investigation. The study group included 180 girls and 180 boys, aged from birth to 18 years. To assess the correlation between the degree of development of the paranasal sinuses and the growth of the cranium, standard anthropometric points on the skull and strictly defined dimensions of the height, length, width, and volume of right and left maxillary sinuses were used. In the study group, both in girls and boys, a statistically significant positive correlation was found at the significance level of *p* < 0.0001 between: the height, length, width and volume of right and left maxillary sinuses, and cranial maximum length (glabella-opisthocranion), its maximum width (euryon-euryon), height (basion-bregma) and the length of the cranial base (basion-nasion) and the dimension of the subspinale-opisthocranion in children. Our study showed a statistically significant positive correlation between the development of maxillary sinuses and the growth of the cranium in children.

## Introduction

Functionally and anatomically, there are two main parts of the cranium: the neurocranium and the viscerocranium. The neurocranium consists of the calvaria and the cranial base. This division has both functional and developmental significance. The neurocranium surrounds and protects the brain, whereas the viscerocranium forms the skeleton of the face. The development of the brain has a decisive influence on the growth of the calvaria. The development of the cranial base is primarily responsible for the elongation of the cranium. However, the growth of the viscerocranium is related to the development of facial organs and structures^1,2^.

Maxillary sinuses are the largest paranasal sinuses, they constitute a large space located inside the jaws and are normally filled with air^3,4^. The development of maxillary sinuses begins already in fetal life and continues after birth^1,4^. In the following years of life, maxillary sinuses increase in size pneumatizing the jaws^3,5,6^.

There are available studies assessing the development of maxillary sinuses in relation to the age of children^7–11^. The studies conducted so far have estimated the development of maxillary sinuses in reference to the development of the viscerocranium. These studies demonstrated a statistically significant correlation between the dimensions of maxillary sinuses and defined anthropometric measurements of the viscerocranium^12^. Przystańska at al. analyzed the development of maxillary sinuses in relation to the following anthropometric measurements: zygion - zygion, zygomaxillare - zygomaxillare, nasion - prosthion, nasospinale - prosthion and nasospinale - P (measurement not found in craniometry)^12^. However, according to our knowledge there are no publications analyzing the development of maxillary sinuses in relation to the development of the cranium, i.e. both the viscerocranium and the neurocranium. The only available study is the one analyzing the development of maxillary sinuses with reference to head circumference in human fetuses^13^.

## Aim

The aim of the study was to analyze the relationship between the defined dimensions of maxillary sinuses and strictly determined anthropometric measurements of the cranium, including both the viscerocranium and the neurocranium in children.

## Materials and methods

The study was retrospective and was conducted at the Department of Diagnostic Imagining, Polish Mother’s Memorial Hospital - Research Institute. The study comprised a group of 360 children, 180 girls and 180 boys, aged from birth to 18 years, who underwent head CT and met the inclusion criteria. All examinations were performed with a 256-row Philips Brilliance computed CAT scanner. All children’s parents and/or legal guardians gave informed consent for head computed tomography investigation and its use for scientific research. This study followed the tenets outlined in the Declaration of Helsinki and was approved by the Bioethics Committee of the Medical University of Lodz, No: RNN/169/18/KE of May 15, 2018. Due to population differences in the growth rate and size of the skull and maxillary sinuses in particular periods of life, the number of children for each year of life was 20 and was the same for girls and boys. The criterion for including patients in the study was correctly performed head CT with all anthropometric points defined in the study. The exclusion criteria were the following: disease of the paranasal sinuses or pathology within them, condition after head surgery, congenital or acquired developmental defect, genetic or metabolic disease, history of growth disorders, traumatic cranial bone changes, active neoplastic process, cranial deformation, bleeding into the central nervous system, artifacts that preventing proper to assessment of the skull and paranasal sinuses.

### Anthropometric measurements of the cranium

To assess the relationship between specific cranial anthropometric measurements and the degree of paranasal sinuses development, there were used standard, precisely defined anthropometric points on the skull (Table 1). The following cranial measurements were taken between the defined anthropometric points^14–17^:

1. Maximum cranial length (g-op): The straight-line distance from glabella (g) to opisthocranion (op) in the median plane (mid-sagittal plane).
2. Maximum cranial breadth (eu-eu): The maximum width of the skull perpendicular to the median plane (mid-sagittal plane) wherever it is located except for the inferior temporal line and the immediate area surrounding the latter (i.e. the posterior roots of the zygomatic arches and supramastoid crest).
3. Cranial height or basion-bregma height (ba-b): The straight distance from the lowest point on the anterior margin of the foramen magnum - basion (ba) to bregma (b).
4. Cranial base length (ba-n): The straight distance from nasion (n) to basion (ba).
5. Subspinale-opisthocranion length (ss-op): The straight distance from subspinale (ss) to opisthocranion (op).
6. Bizygomatic breadth (zy-zy): The straight distance between most lateral points on both zygomatic arches.
7. Zygomaxillare-zygomaxillare breadth (bimaxillary breadth) (zm-zm): The breadth across the maxillae, from the left to right zygomaxillare (zm).
8. Glabella-subspinale height (g-ss): The straight distance from glabella (g) to subspinale (ss).
9. Basion-subspinale length (ba-ss): The straight distance from basion (ba) to subspinale (ss).

**Fig. 1:**
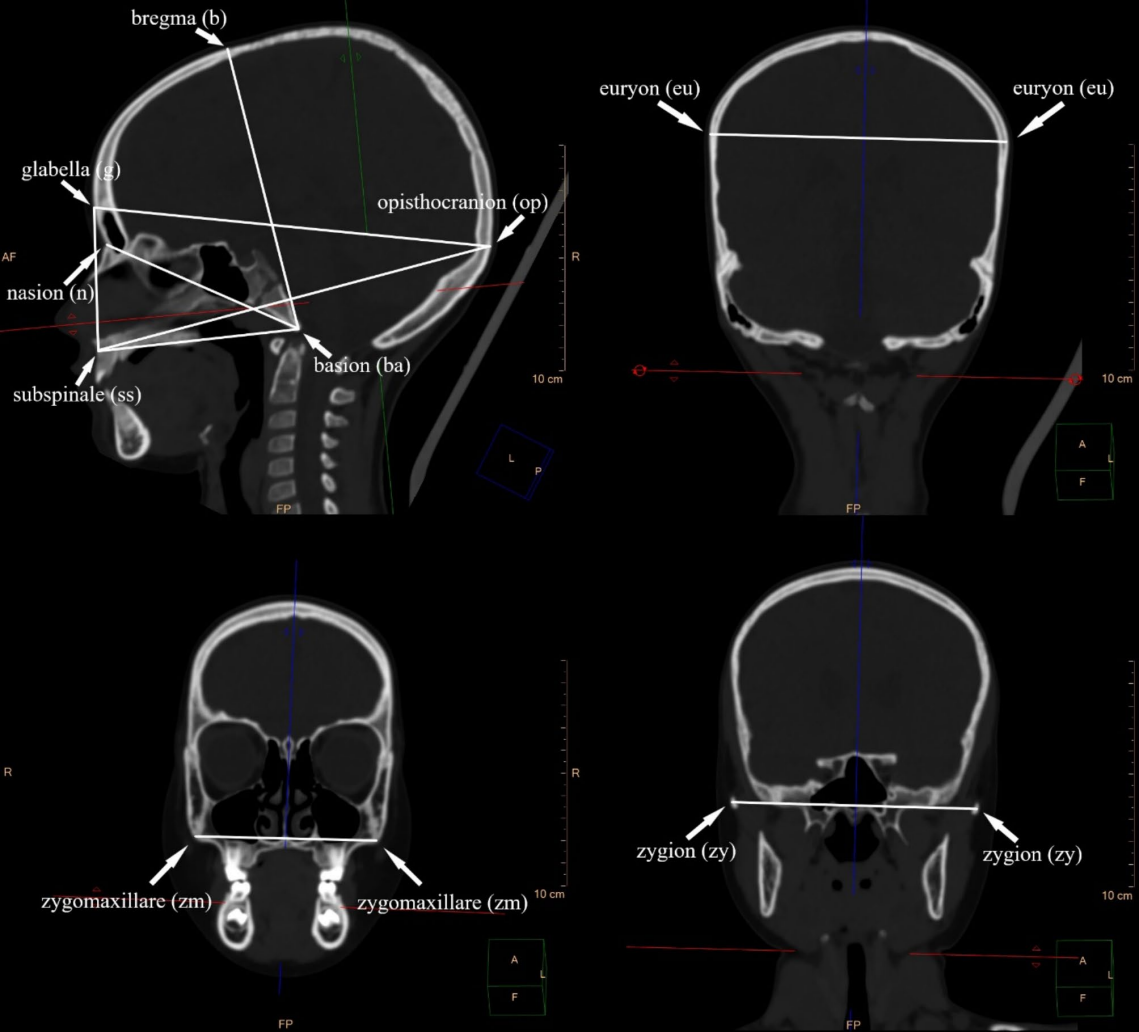
Computed tomography of the head in children demonstrating cranial landmarks and measurements cranium used in the study.

The analyzed points and anthropometric measurements are presented in Fig. 1.

**Table 1:** Anatomical landmarks used in the present study including abbreviations and definitions^14–17^.

Landmark	Definition
Bregma (b)	Most posterior point on the border of the frontal bone in the median plane (mid-sagittal plane), at the intersection of the coronal and sagittal sutures. The latter may diverge from the midline here, however, and should not then be followed (metopic (frontal) sutures should be disregarded). In cases where the most anterior segment of the sagittal suture deflects to one side, the point of the junction of the two sutures must be projected. In cases of asymmetry of the coronal suture, the general course of the suture as a whole should be lightly drawn, and the bregma established on this. In the case of the presence of an open anterior fontanelle, a straight extension of the sagittal suture is drawn across the forehead while a similar connection is drawn between the two sections of the coronal suture. The point should mark the limits of the frontal and parietal segments of the vault generally, not minor sutural variations.
Basion (ba)	The point at which the anterior border of the foramen magnum is intersected by the median plane (mid-sagittal plane). In rare cases the determination of the position of basion may be made difficult by a thickening of the anterior margin. In determination of the height of the cranium, basion is positioned the most inferior point on the anterior border of the foramen magnum is intersected by the median plane.
Euryon (eu) (even)	The most laterally positioned point on the side of the neurocranium. Euryon always falls on either the parietal bone or on the upper portion of the temporal bone and may be determined only by measuring maximum cranial breadth. The area of the root of the zygomatic arch, the supra-mastoid crest, and the entire adjacent region above the external auditory meatus, which sometimes exhibit excessive symmetrical lateral expansion, should be avoided when determining the position of euryon.
Glabella (g)	The most anteriorly projecting point in the median plane (mid-sagittal plane) at the lower margin of the frontal bone, which lies above the nasal root and between the superciliary arches (Frankfort horizontal plane). The point of glabella is depressed between the confining bony ridges and is often delineated superiorly by a shallow gutter or a transversely running indentation on the surface of the frontal bone. In the case of absence of a glabellar prominence, the point should be located at the intersection of the line connecting the superciliary arches with the median plane. If the eyebrow arches are not accentuated, the point should be placed at the intersection of the median plane and the line connecting the highest points located on the upper edge of the eye sockets. On children’s skulls, the glabella is not always the most anterior point.
Nasion (n)	The point of intersection of the frontonasal suture and the median plane (mid-sagittal plane).
Opisthocranion (op)	The most distant point posteriorly from glabella (g) on the occipital bone, located in the median plane (mid-sagittal plane). Opisthocranion almost always falls on the superior squama of the occipital bone, and only occasionally on the external occipital protuberance. Opisthocranion is established by obtaining the measurement of maximum cranial length.
Subspinale (ss)	A point located on the intermaxillary suture, most inferiorly at the base of the anterior nasal spine, at the transition to the anterior surface of the alveolar process of maxilla, in the median plane.
Zygion (zy) (even)	The most laterally positioned point on the zygomatic arches. The position of zygion is defined from the measurement of the greatest distance of the two zygion points.
Zygomaxillare (zm) (even)	The most inferior, anterior point on the zygomaticomaxillary suture, not on the inferior aspect.

### Measurements of the maxillary sinuses

**Fig. 2:**
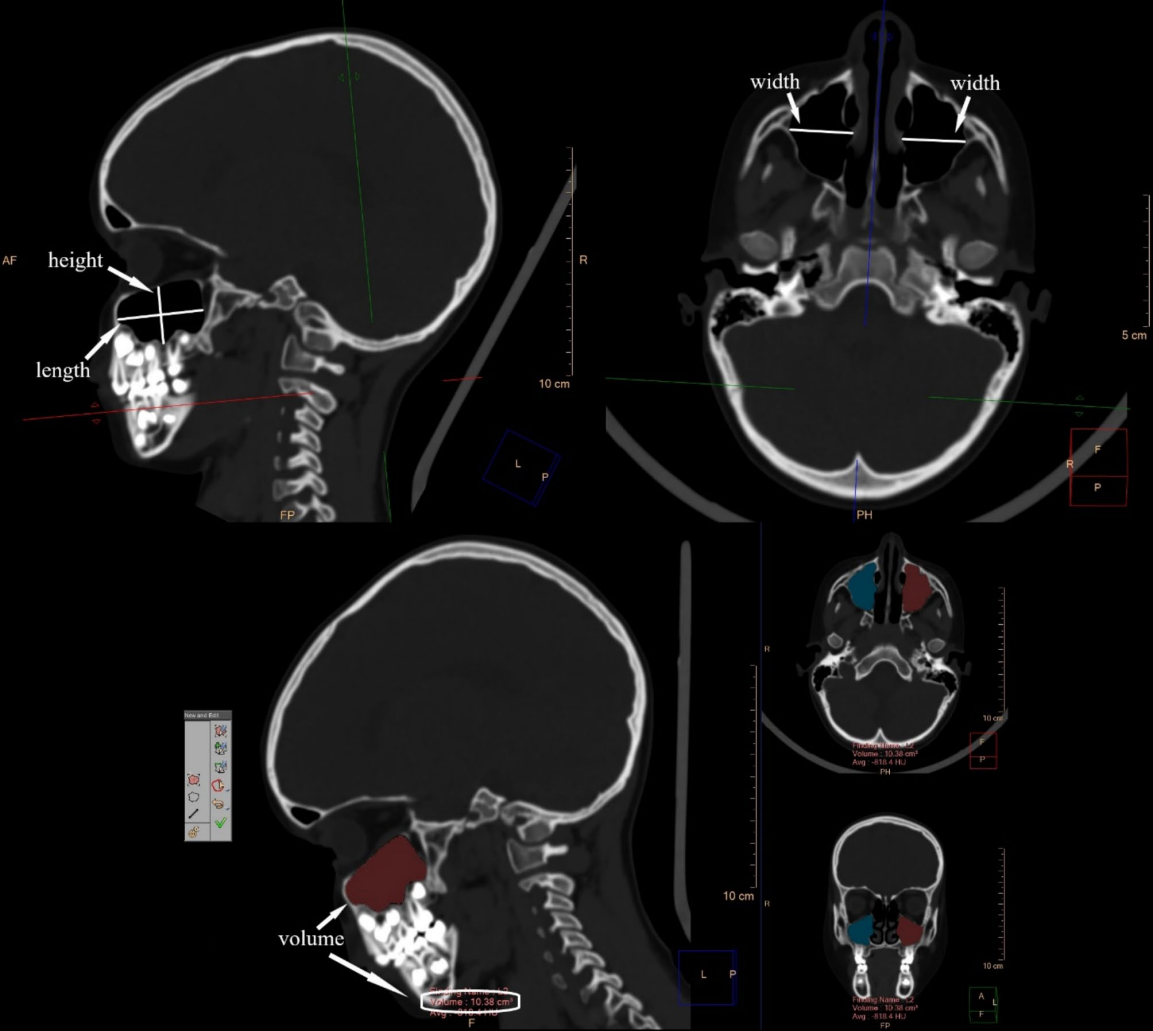
Computed tomography of the head in children showing the measurements of the maxillary sinuses: height, length, width and volume used in the study.

The assessment of maxillary sinuses in each patient included bilateral measurements in three planes: sagittal, frontal and transverse. The maximal vertical diameter of the maxillary sinus perpendicular to the transverse plane was defined as the height of the maxillary sinus. The maximum anteroposterior diameter of the maxillary sinus perpendicular to the frontal plane was defined as the length of the maxillary sinus and the maximum transverse diameter of the maxillary sinus perpendicular to the sagittal plane was defined as the width of the maxillary sinus. The volume of maxillary sinuses was also measured using the IntelliSpace Portal 7.0 program with the “Tumor Tracking” option. All measurements are the internal dimensions of maxillary sinuses taken in the bone window (Fig. 2).

### Statistical analysis

Descriptive statistical values for continuous variables were presented as the mean and standard deviation along with 95% confidence interval (CI). Nominal variables were described as counts. Student’s t-test was used to compare a continuous variable between two groups, and paired student’s t-test was used to compare measurements between left and right side. Correlations between variables were assessed using Pearson correlations. The following division was used to describe the Pearson correlation coefficient: 0.0–0.3 - no correlation, weak; 0.31–0.5 - moderate; 0.51–0.7 - high; 0.71–0.9 - strong; 0.91–1.0 - very strong, full. Scatter plots demonstrate correlations between selected variables. STATISTICA 13.1 (TIBCO Software, Palo Alto, CA, USA) was used to perform statistical analysis. A p value < 0.05 was considered statistically significant.

## Results

The mean age of the study group was 9.00 ± 5.20 years, of girls: 9.01 ± 5.22 years and of boys: 9.00 ± 5.20 years, *p* = 0.9805. The mean values of all analyzed anthropometric measurements of the cranium were higher in boys than in girls. For all analyzed measurements except s-sm and g-ss, this was a statistically significant difference at the significance level of *p* < 0.05 (Table 2). The mean volume, height, length, and width of both right and left maxillary sinuses were larger in boys than in girls, and this difference was not statistically significant, *p* ≥ 0.05 (Table 3). No statistically significant difference was found between the volume (*p* = 0.8094), height (*p* = 0.0964), length (*p* = 0.3361) and width (*p* = 0.4363) of right and left maxillary sinuses.

**Table 2:** Anthropometric measurements of the cranium: in the study group, in girls and in boys.

Cranium measurements	Group (*n* − 360) Mean ± SD (95% Cl)	Girls (*n* − 180) Mean ± SD (95% Cl)	Boys (*n* − 180) Mean ± SD (95% Cl)	p*
g-op [mm]	171.80 ± 11.50 (170.61–173.00)	169.50 ± 10.82 (167.90-171.09)	174.11 ± 11.72 (172.39-175.84)	**0.0001**
eu-eu [mm]	138.17 ± 8.39 (137.30-139.04)	136.31 ± 7.93 (135.14-137.47)	140.03 ± 8.44 (138.79-141.27)	**< 0.0001**
ba-b [mm]	131.40 ± 9.48 (130.41-132.38)	129.23 ± 8.60 (127.97-130.49)	133.56 ± 9.84 (132.11-135.01)	**< 0.0001**
ba-n [mm]	93.63 ± 9.66 (92.63–94.63)	92.06 ± 8.95 (90.74–93.38)	95.20 ± 10.10 (93.72–96.69)	**0.0019**
ss-op [mm]	178.55 ± 13.28 (177.18-179.93)	176.35 ± 12.80 (174.46-178.23)	180.76 ± 13.43 (178.79-182.74)	**0.0015**
zy-zy [mm]	113.70 ± 12.66 (112.39-115.01)	112.29 ± 12.15 (110.50-114.08)	115.11 ± 13.04 (113.19-117.03)	**0.0344**
zm-zm [mm]	78.21 ± 9.42 (77.23–79.19)	77.34 ± 9.19 (75.99–78.70)	79.08 ± 9.58 (77.67–80.49)	0.0806
g-ss [mm]	59.04 ± 7.79 (58.23–59.85)	58.25 ± 7.63 (57.13–59.38)	59.83 ± 7.90 (58.67–60.99)	0.0549
ba-ss [mm]	83.93 ± 8.30 (83.07–84.79)	82.64 ± 7.75 (81.50-83.78)	85.22 ± 8.64 (83.95–86.49)	**0.0031**

p*- girls vs. boys.

Significant values are in bold.

​

**Table 3:** Measurements of the right and left maxillary sinus: in the study group, in girls and in boys.

Maxillary sinus measurement	Right maxillary sinus				Left maxillary sinus			
	Group (*n* − 360) Mean ± SD (95% Cl)	Girls (*n* − 180) Mean ± SD (95% Cl)	Boys (*n* − 180) Mean ± SD (95% Cl)	p*	Group (*n* − 360) Mean ± SD (95% Cl)	Girls (*n* − 180) Mean ± SD (95% Cl)	Boys (*n* − 180) Mean ± SD (95% Cl)	p*
Volume [cm3]	11.03 ± 6.62 (10.34–11.71)	10.66 ± 6.27 (9.74–11.59)	11.39 ± 6.96 (10.37–12.41)	0.2984	11.04 ± 6.64 (10.35–11.73)	10.64 ± 6.31 (9.71–11.56)	11.45 ± 6.95 (10.43–12.47)	0.2464
Height [mm]	27.05 ± 8.89 (26.13–27.98)	26.92 ± 8.89 (25.61–28.23)	27.19 ± 8.92 (25.87–28.50)	0.7779	27.20 ± 8.96 (26.27–28.13)	26.98 ± 8.95 (25.66–28.29)	27.43 ± 9.00 (26.10-28.75)	0.6341
Length [mm]	34.27 ± 6.70 (33.58–34.97)	33.83 ± 6.74 (32.84–34.82)	34.72 ± 6.65 (33.74–35.69)	0.2091	34.19 ± 6.62 (33.51–34.88)	33.70 ± 6.73 (32.71–34.69)	34.68 ± 6.49 (33.73–35.63)	0.1619
Width [mm]	24.36 ± 6.86 (23.65–25.07)	23.97 ± 6.90 (22.96–24.99)	24.74 ± 6.81 (23.74–25.74)	0.2883	24.27 ± 6.82 (23.57–24.98)	23.96 ± 6.89 (22.95–24.98)	24.58 ± 6.77 (23.59–25.58)	0.3908

p*- girls vs. boys.

Analyzing the correlation between the dimensions of right and left maxillary sinuses, respectively, and the anthropometric measurements of the cranium, a statistically significant positive correlation was found at the significance level of *p* < 0.0001 for all analyzed variables in the study group, both for girls and boys.

**Fig. 3:**
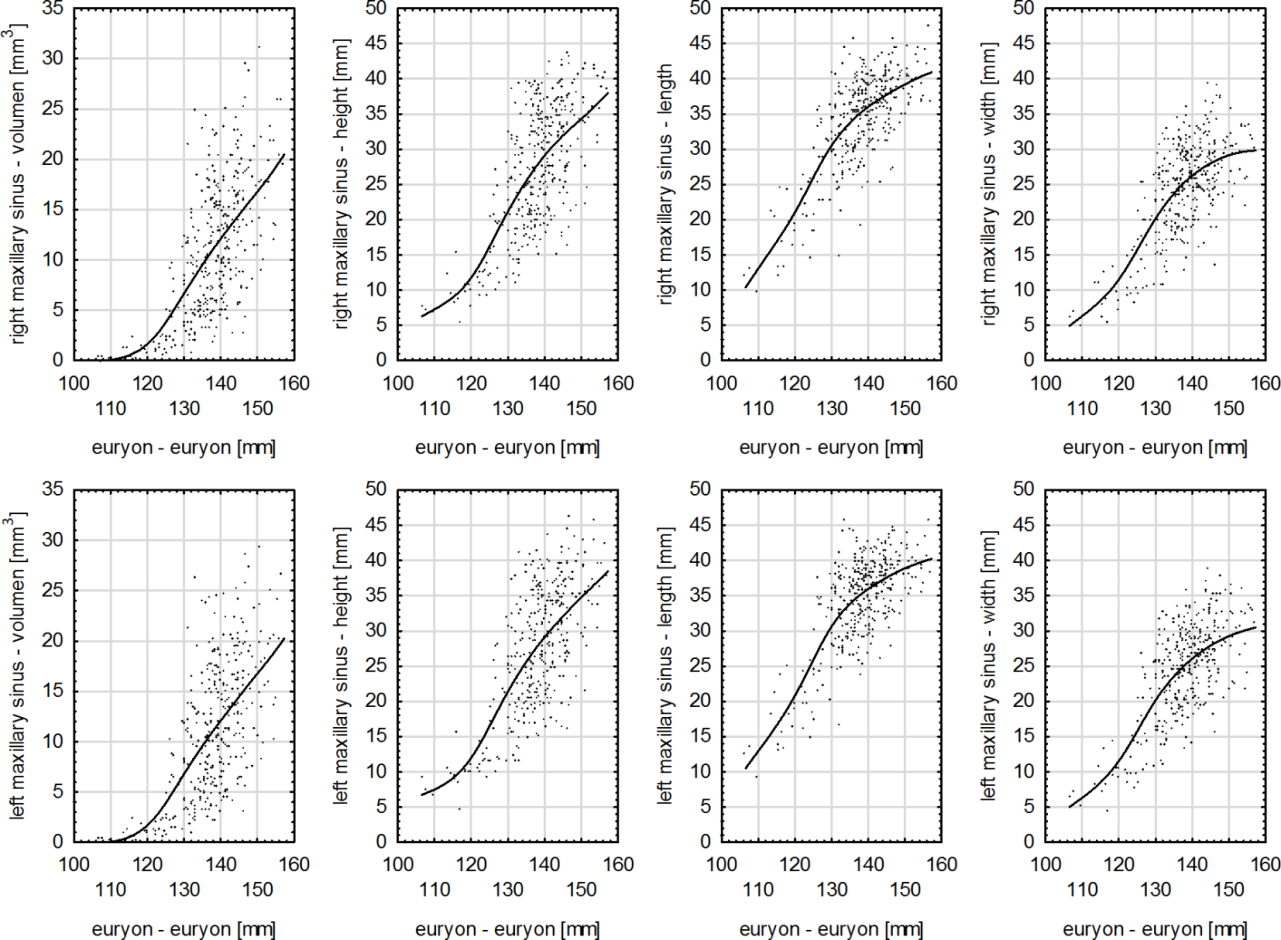
Graphs of the correlations between the measurements of the right and left maxillary sinuses with the maximum cranial width (eu-eu) in the study group (*p* < 0.0001).

**Fig. 4:**
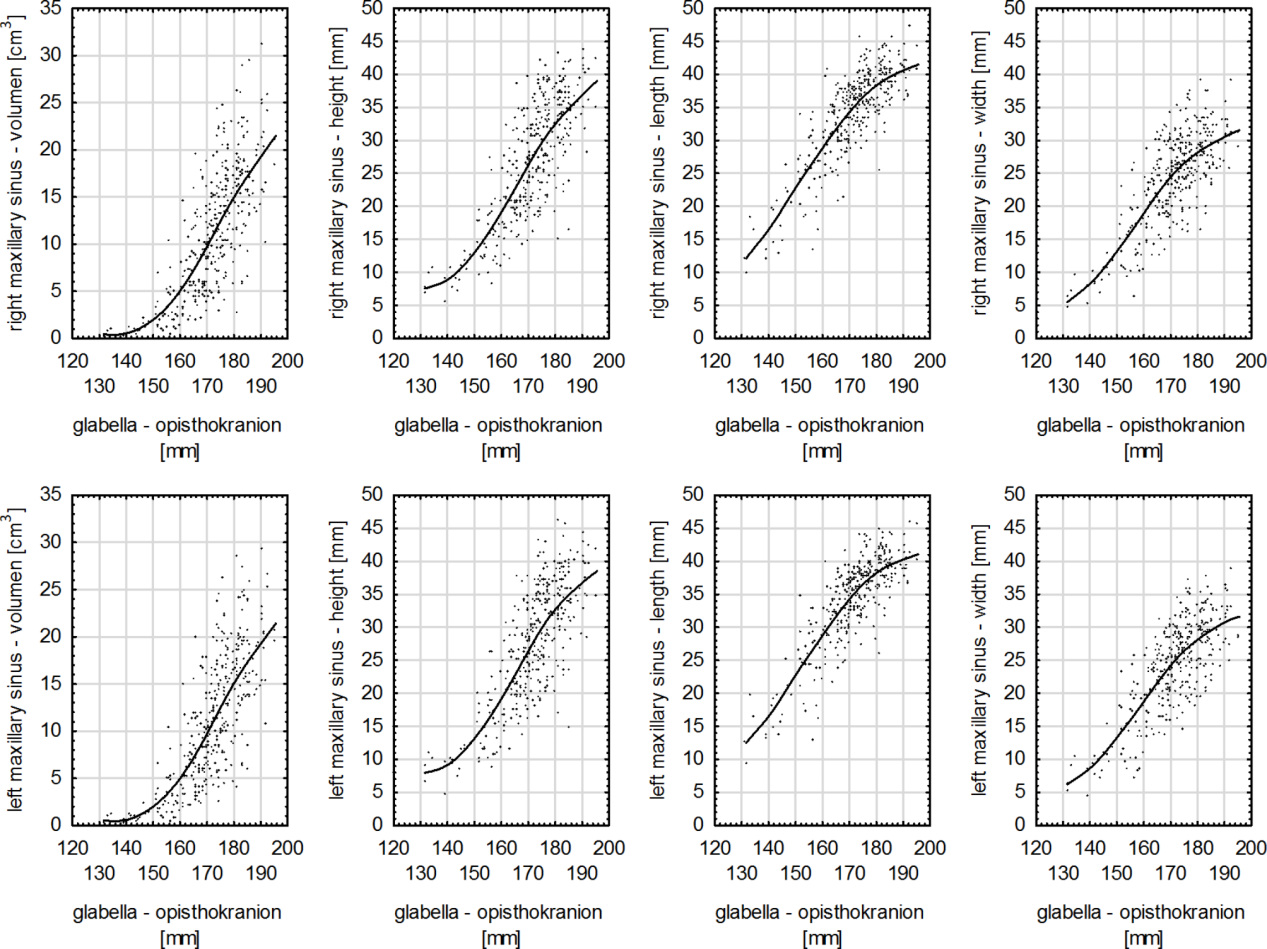
Graphs of the correlations between the measurements of the right and left maxillary sinuses with the maximum cranial length (g-op) in the study group (*p* < 0.0001).

**Fig. 5:**
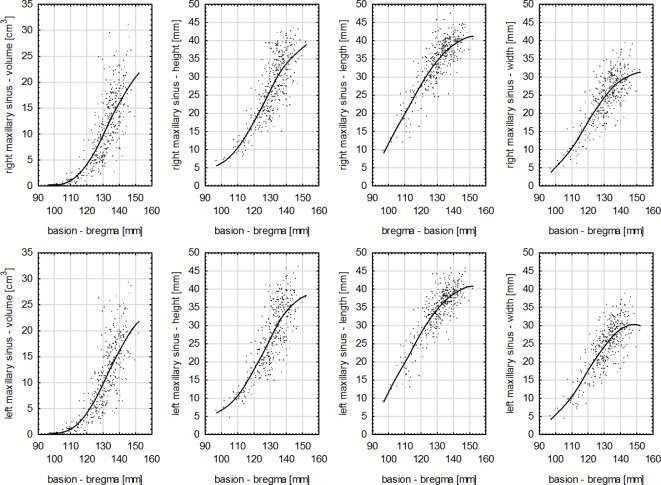
Graphs of the correlations between the measurements of the right and left maxillary sinuses and the cranial height (ba-b) in the study group (*p* < 0.0001).

​ In the study group, the Pearson correlation coefficient, considering all dimensions of right and left maxillary sinuses, had the highest values for the measurement of zm-zm. However, for the lowest values of maxillary sinuses dimensions and the maximal width of the cranium (eu-eu) the correlation was statistically significant and high, and for the length of maxillary sinuses it was strong (Fig. 3). In the case of the dimensions of right and left maxillary sinuses and anthropometric measurements of the cranium: the maximum cranial length (g-op) (Fig. 4), height (ba-b) (Fig. 5) and base length (ba-n) as well as ss-op measurement the correlation was strong for all variables (Table 4). For the remaining anthropometric measurements analyzed in the study group, a strong correlation was obtained between these variables, except for the height of maxillary sinuses and the following measurements: zy-zy, zm-zm and g-ss, for which the correlation was very strong (Table 4).

Analyzing the obtained anthropometric measurements of the cranium depending on the dimensions of right and left maxillary sinuses in girls and boys, respectively, for the cranial height (ba-b) and the maximum cranial width (eu-eu), the values of Pearson correlation coefficients for all variables were higher in boys than in girls. In the group of boys, for the maximum cranial width (eu-eu) a strong correlation was obtained with the height, length and width of right and left maxillary sinuses, whereas for the volume of maxillary sinuses the correlation was high. In the group of girls, the correlation was high for the maximum cranial width (eu-eu) and all dimensions of maxillary sinuses. For the remaining analyzed variables, a strong correlation was obtained, except for the height of maxillary sinuses in girls and the g-ss measurement and in boys for the height of maxillary sinuses and the zy-zy measurement and the height of the left maxillary sinus and the zm-zm measurement for which the correlation was very strong. Moreover, the study showed that for the volume of maxillary sinuses and all cranial measurements except g-ss, the Pearson correlation coefficient was higher in boys than in girls (Table 5).

**Table 4:** Correlations of the dimensions of the right and left maxillary sinus with the dimensions of the cranium in the study group. The Pearson correlation coefficient (r) is provided, p < 0.0001 for all analyzed correlations.

Cranium measurement	Right maxillary sinus				Left maxillary sinus			
	Volume	Height	Length	Width	Volume	Height	Length	Width
g-op	0.74	0.78	0.82	0.76	0.74	0.78	0.82	0.76
eu-eu	0.63	0.68	0.71	0.68	0.62	0.68	0.71	0.68
ba-b	0.76	0.81	0.82	0.79	0.76	0.81	0.83	0.79
ba-n	0.86	0.88	0.89	0.84	0.86	0.87	0.88	0.83
ss-op	0.78	0.81	0.84	0.79	0.78	0.80	0.84	0.79
zy-zy	0.89	0.91	0.88	0.86	0.88	0.91	0.87	0.86
zm-zm	0.90	0.91	0.89	0.89	0.90	0.91	0.88	0.88
g-ss	0.87	0.91	0.85	0.83	0.86	0.91	0.84	0.83
ba-ss	0.82	0.81	0.86	0.78	0.82	0.81	0.86	0.77

## Discussion

In our study, we showed a statistically significant positive correlation between the development of maxillary sinuses and the growth of the cranium, i.e. strictly defined dimensions of maxillary sinuses, and the maximum cranial length (g-op), its maximum width (eu-eu), height (ba-b) and cranial base length (ba-n) and ss-op diameter in children. We also confirmed the existence of a statistically significant positive correlation between specific anthropometric measurements of the viscerocranium and the dimensions of maxillary sinuses. Available studies describe the correlation between the development of maxillary sinuses and the viscerocranium. The study of Przystańska et al. showed that all dimensions of maxillary sinuses significantly correlated with midface parameters analyzed in this study^12^. The Pearson correlation coefficient for individual dimensions of maxillary sinuses and the analyzed cranial measurements most often had the highest values for the zm-zm diameter. It should be noted that the zm-zm diameter is an anthropometric dimension determining the midface width, which mainly covers the jaws. The fact is that maxillary sinuses develop in jaws. At the same time, the development of jaws influences the growth of the viscerocranium^1,3,7^. Literature data clearly demonstrate that the development of the nasal cavity and paranasal sinuses is closely related to the development of the viscerocranium^1,7^. It seems that the developmental distinctiveness of individual parts of the cranium and the resulting division into the viscerocranium, calvaria and the cranial base may have an impact on the lack of studies that analyze the development of maxillary sinuses in relation to the development of the cranium as a whole, and not only the viscerocranium^1^. Our study indicates a strong correlation between the growth of maxillary sinuses and the cranium. It is worth noting that maxillary sinuses begin to develop already in fetal life. After birth they continue to develop. Studies assessing the development of maxillary sinuses depending on age indicate that their quickest growth is observed in the first few years of life. However, in the following years of life, the growth rate of maxillary sinuses decreases^3,7–9,11^. In the study of Lorkiewicz-Muszyńska et al., the growth of maxillary sinuses was observed until the age of 16, and in studies of other authors, it was found that slow pneumatization lasts until the age of 18 or 20 years^7,9,18^. At the same time, the cranium develops most dynamically during the fetal period and in the first years of life after birth. The cranium grows, despite different growth rates of its individual parts, for several years until it reaches its final size^2,19^.

**Table 5:** Correlations of the dimensions of the right and left maxillary sinus with the dimensions of the cranium: in girls and in boys. The Pearson correlation coefficient (r) is provided, p < 0.0001 for all analyzed correlations.

Cranium measurement		Right maxillary sinus				Left maxillary sinus			
		Volume	Height	Length	Width	Volume	Height	Length	Width
Girls	g-op	0.74	0.8	0.83	0.78	0.73	0.8	0.82	0.78
	eu-eu	0.59	0.66	0.66	0.65	0.57	0.64	0.67	0.66
	ba-b	0.75	0.82	0.82	0.79	0.75	0.82	0.82	0.8
	ba-n	0.85	0.88	0.9	0.85	0.84	0.88	0.88	0.85
	ss-op	0.77	0.82	0.85	0.81	0.77	0.82	0.85	0.8
	zy-zy	0.86	0.89	0.84	0.82	0.86	0.9	0.86	0.85
	zm-zm	0.89	0.9	0.88	0.87	0.89	0.9	0.9	0.89
	g-ss	0.88	0.93	0.85	0.83	0.86	0.92	0.83	0.83
	ba-ss	0.79	0.81	0.87	0.8	0.78	0.8	0.86	0.79
Boys	g-op	0.75	0.8	0.82	0.75	0.76	0.78	0.83	0.77
	eu-eu	0.67	0.73	0.77	0.72	0.66	0.73	0.75	0.72
	ba-b	0.78	0.84	0.85	0.83	0.79	0.83	0.86	0.81
	ba-n	0.87	0.9	0.89	0.83	0.88	0.89	0.89	0.82
	ss-op	0.8	0.82	0.83	0.78	0.8	0.8	0.85	0.79
	zy-zy	0.89	0.91	0.88	0.86	0.89	0.92	0.89	0.85
	zm-zm	0.9	0.9	0.88	0.88	0.9	0.91	0.89	0.88
	g-ss	0.86	0.9	0.85	0.83	0.86	0.9	0.84	0.84
	ba-ss	0.84	0.83	0.85	0.78	0.85	0.82	0.86	0.77

The recently available studies concern only the development of maxillary sinuses in relation to head circumference in human fetuses. The study of Farah et al. assessed the development of maxillary sinuses in human fetuses in three dimensions: anteroposterior, vertical and transverse. One of the elements of the analysis was the comparison of the obtained dimensions of maxillary sinuses with the head circumference. The increase in the vertical and transverse dimensions of maxillary sinuses was demonstrated to be synchronized with the head circumference. However, the increase in the anteroposterior dimension was faster in relation to head circumference with a variable rate during intrauterine life^13^. Koppe et al., in their study on maxillary sinuses in primates, analyzed the postnatal growth of maxillary sinuses of orangutans – orang-utan, Pongo satyrus borneensis. The authors showed a strong correlation between the volume of maxillary sinuses and the length of the cranial base (basion to nasion) for both females and males^20^.

There is a consensus among researchers that maxillary sinuses in men are larger than in women. In the studies of Barghouth et al., Bhushan et al., Lorkiewicz-Muszyńska et al., maxillary sinuses in boys were larger than in girls and this difference was not statistically significant^7–9^. Adibelli et al. also reported that maxillary sinuses were larger in boys, but the difference was statistically significant^10^. In our study, we found that the mean value of individual dimensions of maxillary sinuses in boys was higher than in girls and these differences were not statistically significant. Barghouth et al., Bhushan et al., Lorkiewicz-Muszyńska et al. and Adibelli et al. in their research on the development of maxillary sinuses did not show a statistically significant difference between the size of the right and left maxillary sinuses^7–10^. In our study, we also did not demonstrate a statistically significant difference between the analyzed dimensions of the right and left maxillary sinuses.

The role of the paranasal sinuses remains controversial^21^. Without a doubt, however, maxillary sinuses are structures that occur in normal human anatomy. Therefore, knowledge of their development in relation to cranium development may be useful in many areas of medicine. This knowledge is important, among others, when planning cranial surgery, increases the safety of endoscopic procedures of maxillary sinuses and minimizes complications^3,8^. The obtained results, are important because they allow a broader and new perspective on the development of maxillary sinuses and cranium.

Limitations of the study: In our study, we did not perform anthropometric measurements of the mandible, for example the anthropometric gnathion point. This was dictated by the fact that the position of the mandible during CT examination in children is different. Some children have their mouths closed; others more or less opened. Thus, the location of the points on the mandible depends to a greater or lesser extent on the position of the mandible, which could significantly affect the accuracy of anthropometric measurements.

## Conclusions

In our study, we showed a statistically significant positive correlation between the volume, height, length and width of the right and left maxillary sinuses, and the maximum length of the cranium (g-op), its maximum width (eu-eu), height (ba-b) and the length of the cranial base (ba-n) and ss-op dimension in children. We found that for the height of the cranium (ba-b) and its maximum width (eu-eu), the values of Pearson’s correlation coefficients for all measurements of maxillary sinuses were higher in boys than in girls. Furthermore, we also confirmed the correlation between anthropometric measurements of the viscerocranium and the dimensions of maxillary sinuses.

## Data Availability

The data that support the findings of this study are available from the corresponding author: Przemysław Kiciński, e-mail: kicinskiprzemko@gmail.com or przemyslaw.kicinski@umed.lodz.pl.
